# The American Text Book of Prosthetic Dentistry

**Published:** 1897-11

**Authors:** 


### Bibliographical.

THE AMERICAN TEXT BOOK OF PROSTHETIC DENTISTRY. By eminent American authorities. Editor : Charles J. Essig, M. D., D. D. S., Professor of Mechanical Dentistry and Metallurgy in the Dental Department of the University of of Pennsylvania. Publishers : Lea Brothers & Co., Philadelphia and New York, 1896.

This work, edited by the well known author of Dental Metallurgy, and consisting of valuable contributions by himself and other eminent practitioners and teachers, is composed of 760 pages of text which are illustrated by 983 engravings, many of which are original, clear and instructive, and will be a valuable aid to the student and practitioner of dentistry. The arrangements of the subjects treated, which include all that this branch of dental art implies, is admirable and the publishers are to be commended for the attractive style in which this treatise is printed. The contents consist of the following subjects all of which are treated in a clear and concise manner suitable for a text book.

The articles by the editor , Dr. Essig, are upon the following subjects .

"The Dental Laboratory; Its Equipment and Management," Metals and Alloys Used in Prosthetic Dentistry," "Moulding and Carving Porcelain Teeth," "English Tube Teeth; Their Use in Plate, Crown and Bridge-work," "Vulcanized Rubber as a Base for Artificial Dentures," "Hygienic Relations and Care of Artificial Dentures."

The subjects treated of by Henry H. Burchard, M. D., D. D. S., are :

"Upon the Preparation of the Mouth; Choice of Material and Type of Denture," 'Taking Impressions of the Mouth," "Making of Models and Their Preparation," "Dies, Counter-dies and Moulding," "Swaged Metallic Plates," "Selecting and Fitting the Teeth; Attachment to the Plate; Finishing," "Artificial Crowns," "The Assemblage of United Crowns (Bridge-work)."

C. L. Goddard, D. D. S,, treats of "Principles of Metal Work" and "Cast Dentures of Aluminum and Fusible Alloys."

Grant Molyneaux, M. D., D. D. S., upon, "The 'Bite' or Occlusion."

Ambler Tees, D. D. S., of "Continuous Gum Dentures."

W. W Evans, M. D., D. D. S., of 'Celluloid and Zylonite."

Alton Howard Thompson, D. D. S., of "The Temperaments and the Temperamental Characteristics of the Teeth in Relation to Dental Prosthesis."

Rodrigues Ottolengui, M. D. S., of "Palatal Mechanism."

All of these contributors have presented valuable and instructive articles, well deserving the careful attention and study of students and practitioners.

